# Human Cytomegalovirus Glycoprotein UL141 Targets the TRAIL Death Receptors to Thwart Host Innate Antiviral Defenses

**DOI:** 10.1016/j.chom.2013.02.003

**Published:** 2013-03-13

**Authors:** Wendell Smith, Peter Tomasec, Rebecca Aicheler, Andrea Loewendorf, Ivana Nemčovičová, Eddie C.Y. Wang, Richard J. Stanton, Matt Macauley, Paula Norris, Laure Willen, Eva Ruckova, Akio Nomoto, Pascal Schneider, Gabriele Hahn, Dirk M. Zajonc, Carl F. Ware, Gavin W.G. Wilkinson, Chris A. Benedict

**Affiliations:** 1 The La Jolla Institute for Allergy and Immunology, 9420 Athena Circle, La Jolla, CA 92037, USA; 2 Institute of Infection and Immunity, Department of Medical Microbiology, School of Medicine, Cardiff University, Cardiff CF14 4XN, UK; 3 Laboratory of Molecular Immunology, Infectious and Inflammatory Diseases Research Center, Sanford-Burnham Medical Research Institute, 10901 North Torrey Pines Road, La Jolla, CA 92037, USA; 4 Biochemistry Department, University of Lausanne, 1066 Epalinges, Switzerland; 5 Regional Centre for Applied Molecular Oncology (RECAMO), Masaryk Memorial Cancer Institute, Zluty Kopec 7, 65653 Brno, Czech Republic; 6 Institute of Microbial Chemistry (Bikaken), 3-14-23 Kamiosaki, Shinagawa-ku, Tokyo 141-0021, Japan; 7 MVZ Dresden Labor Möbius Quasdorf, Bayreuther Strasse 30, 01187 Dresden, Germany

## Abstract

Death receptors (DRs) of the TNFR superfamily contribute to antiviral immunity by promoting apoptosis and regulating immune homeostasis during infection, and viral inhibition of DR signaling can alter immune defenses. Here we identify the human cytomegalovirus (HCMV) UL141 glycoprotein as necessary and sufficient to restrict TRAIL DR function. Despite showing no primary sequence homology to TNF family cytokines, UL141 binds the ectodomains of both human TRAIL DRs with affinities comparable to the natural ligand TRAIL. UL141 binding promotes intracellular retention of the DRs, thus protecting virus infected cells from TRAIL and TRAIL-dependent NK cell-mediated killing. The identification of UL141 as a herpesvirus modulator of the TRAIL DRs strongly implicates this pathway as a regulator of host defense to HCMV and highlights UL141 as a pleiotropic inhibitor of NK cell effector function.

## Highlights

► Human cytomegalovirus (HCMV) blocks cell-surface expression of TRAIL death receptors ► HCMV glycoprotein UL141 binds TRAIL death receptors directly ► UL141 inhibits TRAIL-mediated killing of infected cells ► UL141 inhibits TRAIL-dependent, NK cell effector function

## Introduction

Tumor necrosis factor (TNF) family cytokines are crucial in providing protection against virus infections through their regulation of cell death and survival (Benedict, 2003). TNF family cytokines mediate direct antiviral activity in infected cells but also function to maintain immune homeostasis by limiting tissue damage, largely by inducing apoptosis in effector cells after infection is controlled. In turn, viruses encode mechanisms to promote cell survival, facilitating successful replication and transmission. The large DNA herpesviruses all establish lifelong infection, and consequently they employ many strategies to modulate cellular apoptotic signaling pathways. These range from restricting ligand-receptor interactions to blocking caspase activation and activating prosurvival pathways (Loewendorf and Benedict, 2010; Mocarski et al., 2012; Rahman and McFadden, 2006), ultimately impacting the duration of infection and the magnitude of downstream immune responses.

Human cytomegalovirus (HCMV, human herpesvirus 5), the prototype member of the *Betaherpesvirinae*, exhibits a ubiquitous distribution worldwide and seroprevalence varies with socioeconomic status, age, and geography. While infection is commonly asymptomatic in healthy individuals, HCMV can cause severe morbidity and mortality in both the immunocompromised and naive; vaccine development is a high priority (Schleiss, 2007). Moreover, persistent/latent infection is associated with cancer and multiple autoinflammatory disorders (Dziurzynski et al., 2012) (Baryawno et al., 2011; Cobbs et al., 2002; Söderberg-Nauclér, 2006; Streblow et al., 2008), as well as earlier all-cause mortality (Simanek et al., 2011). HCMV has become a paradigm for immune modulation, and its study is proving particularly insightful in characterizing the key mechanisms responsible for regulating human NK cell function. HCMV strategically restricts cell-surface expression of key immune-activating proteins, including TNFR1-, MHC-I-, and natural killer (NK)-cell-activating ligands (Loewendorf and Benedict, 2010; Loureiro and Ploegh, 2006; Wilkinson et al., 2008). UL141 is a type I transmembrane glycoprotein encoded within the the right hand end of the UL (UL/*b*′) region of the HCMV genome that was lost from the commonly-used laboratory strains AD169 and Towne during in vitro passage (Cha et al., 1996). UL141 is recognized to have potent NK cell evasion function that acts by suppressing cell-surface expression of CD155, the ligand for the NK-cell-activating receptors DNAM1 and CD96 (Tactile) (Prod’homme et al., 2010; Tomasec et al., 2005; Xu and Jin, 2010). Moreover, UL141 has also recently been shown to be instrumental in targeting the alternative DNAM1-activating ligand, CD112 (Prod’homme et al., 2010).

In humans, TNF-related apoptosis-inducing ligand (TRAIL/TNFSF10) binds the death receptors (DRs) TRAIL-R1 (DR4) and TRAIL-R2 (DR5). TRAIL also binds additional receptors that do not encode death domains, including TRAIL-R3, TRAIL-R4 and the secreted osteoprotegerin (OPG) receptor; although sometimes referred to as “decoy receptors,” their roles in regulating TRAIL signaling are poorly understood (Ashkenazi and Dixit, 1999; Emery et al., 1998). Clustering of the TRAIL DRs through interaction with their cognate ligand results in their oligomerization, inducing formation of the death-inducing signaling complex (DISC) consisting primarily of FADD and procaspase-8 or procaspace-10 and, ultimately, caspase activation and cellular apoptosis (Bodmer et al., 2000). NK cells produce significant levels of TRAIL when activated by interferons (Sato et al., 2001; Takeda et al., 2001), utilizing it as an effector molecule to kill tumor cells (Feng et al., 2010; Hallett et al., 2008; Smyth et al., 2001). In addition to its established antitumor properties, TRAIL signaling plays a multifaceted role in antiviral immunity, directly targeting infected cells through proapoptotic mechanisms while commensurately regulating immune effectors (Cummins and Badley, 2009). Notably, infection of fibroblasts with HCMV laboratory strain AD169 induces TRAIL-DR expression and sensitizes them to TRAIL killing (Sedger et al., 1999), and HCMV can also induce TRAIL expression in infected dendritic cells (DCs) (Raftery et al., 2001).

HCMV has acquired multiple strategies to counter the TNFR superfamily, including inhibiting the expression and/or signaling by the TNFR-1 and Fas DRs (Baillie et al., 2003; Jarvis et al., 2006; McCormick et al., 2003; Skaletskaya et al., 2001b). Paradoxically, HCMV also actively promotes upregulation of TNFR-1 via the latency-associated UL138 protein (Le et al., 2011; Montag et al., 2011). Moreover, UL144 is an ortholog of HVEM (TNFRSF14) that binds the coinhibitory receptor BTLA and potently inhibits T cell activation (Benedict et al., 1999; Cheung et al., 2005). Here we show that UL141 binds multiple TRAIL DRs through ectodomain interactions, suppressing their cell-surface expression and protecting cells from TRAIL and NK-cell-mediated killing, identifying it as a herpesvirus protein that directly target TRAIL receptors. We propose the TRAIL DR-UL141 interaction represents a countermeasure HCMV has evolved in response to host immune pressure, and further highlights the UL138-144 gene cluster as focused upon targeting the TNFR superfamily.

## Results

### Low-Passage HCMV Strains Inhibit Cell-Surface Expression of the TRAIL Death Receptors

**Figure 1:**
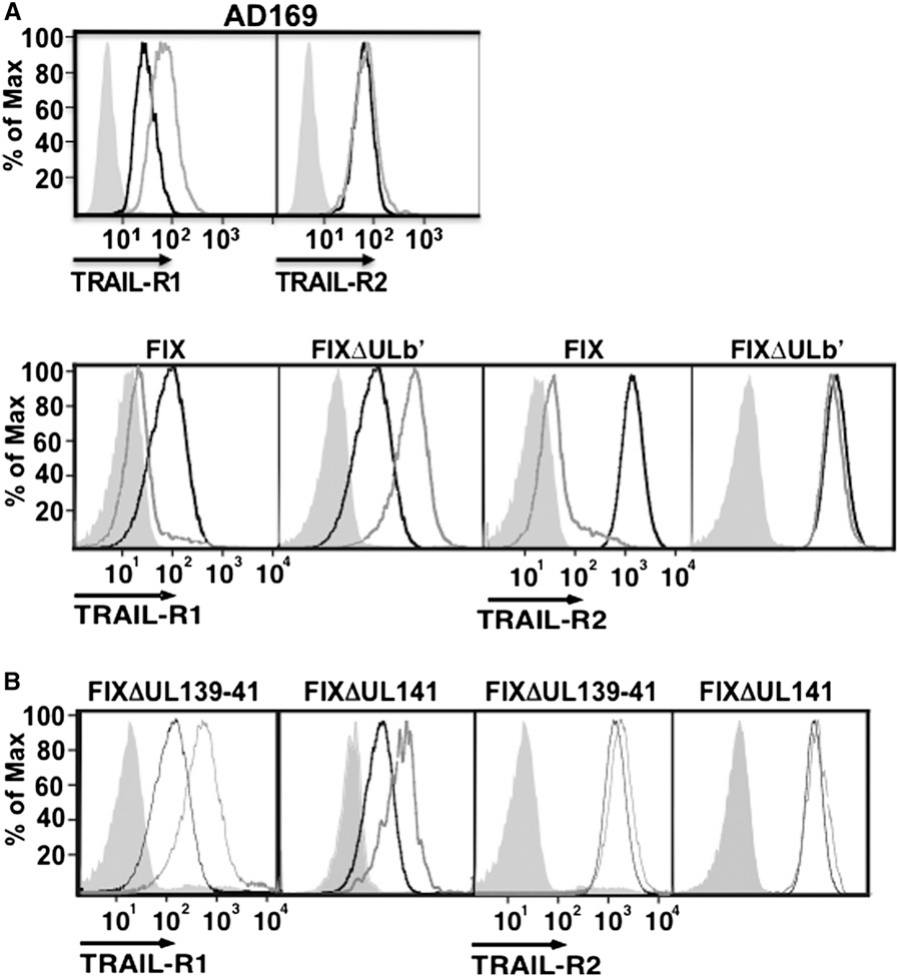
HCMV Restriction of TRAIL DR Cell-Surface Expression Requires UL141 Neonatal human dermal fibroblasts (NHDFs) were infected with the AD169 or FIX strains of HCMV or various deletion mutants at a multiplicity of infection (MOI) of ∼2, and cell-surface levels of TRAIL-R1 and TRAIL-R2 were analyzed 72 hr later by flow cytometry. Black histograms, mock infected; gray histograms, HCMV infected; shaded histograms, isotype control staining of HCMV-infected cells. Similar results were seen in four to six experiments, and isotype control staining for mock-infected cells was either similar to or slightly lower than that displayed for binding to infected cells (≤2× MFI differences). See also Figure S1.

HCMV is known to inhibit signaling by DRs belonging to the TNFR superfamily (e.g., TNFR-1 and Fas) (Baillie et al., 2003; Jarvis et al., 2006; McCormick et al., 2003; Skaletskaya et al., 2001b). However, HCMV isolates that have been passaged extensively in cultured fibroblasts (e.g., the AD169 strain) can differentially alter TNFR expression due to the loss of specific immune modulatory proteins (Le et al., 2011; Montag et al., 2011). Consequently, to address whether infection with HCMV would target the TRAIL DRs, we analyzed fibroblasts infected with distinct viral strains for their cell-surface expression. The high-passage laboratory strain AD169 was used for infection (variant ATCC), as well as the FIX strain of HCMV (originally VR1814), which has been subjected to limited in vitro passage and whose genome is available as an infectious clone in a bacterial artificial chromosome (BAC) (Hahn et al., 2002; Murphy et al., 2003). In contrast to AD169, FIX induced dramatic downregulation of both TRAIL DRs from the cell surface (Figure 1A). The function was ascribed to a de novo FIX-encoded gene product, as inhibition of DR expression was ablated by UV irradiation of input virus (data not shown). Low-passage HCMV strains therefore encode a function that downregulates cell-surface expression of TRAIL-R1 and TRAIL-R2 that has been lost from the laboratory strain AD169.

### UL141 Is Implicated in the Inhibition of TRAIL-R2 Expression

In addition to other defects, strain AD169 has suffered a spontaneous 15 kb deletion from the UL/*b*′ region during passage in vitro (Cha et al., 1996). Consequently, we utilized a HCMV mutant generated in the FIX-BAC deleted in the majority of the UL/*b*′ sequence (FIXΔUL/b′ [Hahn et al., 2004]) to test for the ability to restrict TRAIL DR expression. The UL/*b*′ region contains ≥21 genes that are dispensable for viral replication in fibroblasts (Gatherer et al., 2011). FIXΔUL/b′ could not restrict cell-surface expression of either TRAIL DR, indicating that an HCMV gene contained within this region was required for their inhibition (Figure 1A). Notably, cell-surface expression of TRAIL-R1 was significantly increased after FIXΔUL/b′ infection, and this was consistent with enhanced messenger RNA (mRNA) expression levels seen for this DR in HCMV infected fibroblasts (Figure S1A available online).

Screening through the UL/*b*′ region with a panel of pre-existing FIX BAC deletion mutants (Hahn et al., 2004) ruled out UL128, UL129, UL130, UL131a, UL132, UL148a-d, C-orf23, C-orf25, and C-orf26 in regulating the TRAIL DRs (data not shown). A FIXΔ139-141 mutant was then constructed, and, when tested, this mutant was incapable of inhibiting TRAIL DR expression, with TRAIL-R1 being commensurately upregulated on the cell surface similar to that seen with FIXΔUL/b′ (Figure 1B). Construction of a FIXΔUL141 mutant then revealed that FIX lacking an intact UL141 open reading frame (ORF) was incapable of downregulating cell-surface expression of the TRAIL DR (Figure 1B). Taken together, these results show that UL141 is required to restrict cell-surface expression of TRAIL-R1 and TRAIL-R2 in HCMV-infected cells.

**Figure 2:**
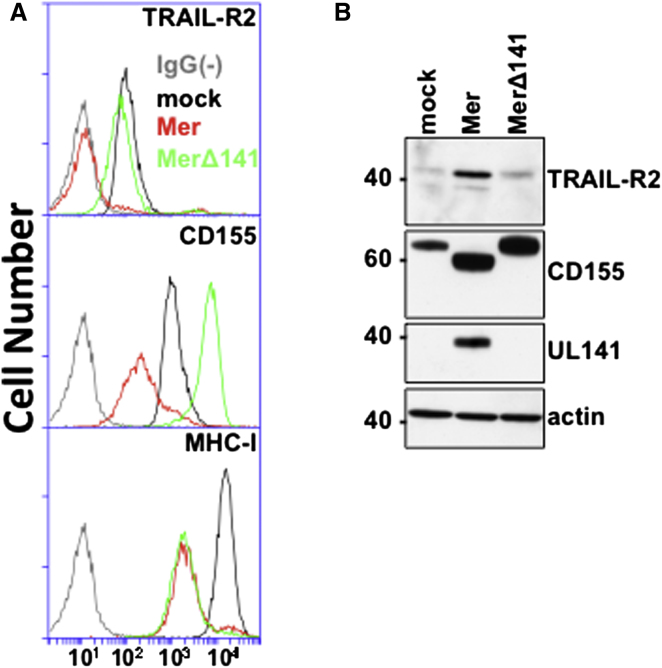
TRAIL-R2 Expression in HCMV-Infected Cells Human foreskin fibroblasts (HFFs) were infected (72 hr, MOI = 20) with HCMV Merlin (Mer) or MerlinΔUL141 (MerΔ141) and analyzed for TRAIL-R2 expression by (A) flow cytometry and (B) Western blot. IgG(–), isotype control antibody staining. Results are representative of six to ten performed experiments. See also Figure S2.

A high level of sequence variation is present in HCMV clinical isolates and cultured strains, although it is not evenly distributed throughout the genome, and this variability has been shown to impact immune evasion functions (Prod’homme et al., 2012). To further examine and confirm the role of UL141 in regulating TRAIL-R1 and TRAIL-R2 expression, we also specifically deleted the gene from HCMV strain Merlin using BAC technology. The Merlin BAC is currently accepted to be the most “genetically intact” cloned HCMV genome in the field, and as the FIX BAC was deleted for the IRS-US6 locus during its construction, the use of Merlin also provided additional confidence in results obtained with FIX. Consistent with previous findings, UL141 was required for downregulation of CD155 by strain Merlin but not for inhibition of MHC-I (Figure 2A). Whereas deletion of UL141 from the FIX strain resulted in restoration of TRAIL-R2 level to those seen in uninfected cells (Figure 1B), in the Merlin strain restoration was never complete, albeit expression levels returned to >90% seen in mock-infected cells. This modest, but reproducible, difference between ΔUL141 mutants of FIX and Merlin suggest potential UL141-independent regulation of the TRAIL DRs by HCMV may exist, and this is currently being explored.

### Expression Kinetics of UL141

Suppression of TRAIL-R2 cell-surface expression by HCMV could be detected as early as 24 hr after infection, yet it became more marked as the infection progressed through 48 and 72 hr (Figure S1D). In strain FIX, UL141 is encoded by a single abundant transcript initiated 213 bases upstream of the start codon and extending to 39 bases downstream of the stop codon (Figure S1B), compatible with recent transcriptional mapping data for UL141 in strain Merlin (Gatherer et al., 2011). Consistent with the kinetics of TRAIL-R2 downregulation, strain FIX UL141 was found expressed as an early-late gene product (Figure S1E), increasing in abundance dramatically throughout the viral replication cycle (Figure S1C).

### Fate of TRAIL-R2 in HCMV-Infected Cells

We have previously shown that UL141 restricts the cell-surface expression of CD155 and CD112, two NK-cell-activating ligands belonging to the nectin/nectin-like family of proteins. Notably, the mechanisms by which UL141 modulates these two host cell proteins are quite distinct. UL141 sequesters CD155 in the endoplasmic reticulum (ER) of HCMV-infected cells (Tomasec et al., 2005) yet promotes the proteasome-dependent degradation of CD112 (Prod’homme et al., 2010). To gain insight into what mechanism(s) of action might be utilized by UL141 to target TRAIL-R2, we analyzed strain Merlin-infected fibroblasts by western blot. Notably, fibroblasts infected with Merlin showed demonstrably higher total cellular levels of TRAIL-R2 when compared to uninfected cells or cells infected with MerΔUL141 (Figure 2B). A similar pattern of restricted cell-surface expression, but enhanced total cellular expression, of TRAIL-R2 was also observed in epithelial cells infected with HCMV. In total, these data indicate that while UL141 functions to inhibit cell-surface expression of TRAIL-R2 in HCMV-infected cells, it appears to promote the accumulation of this DR in an intracellular compartment.

### UL141 Is Sufficient to Restrict TRAIL Death Receptor Cell-Surface Expression

**Figure 3:**
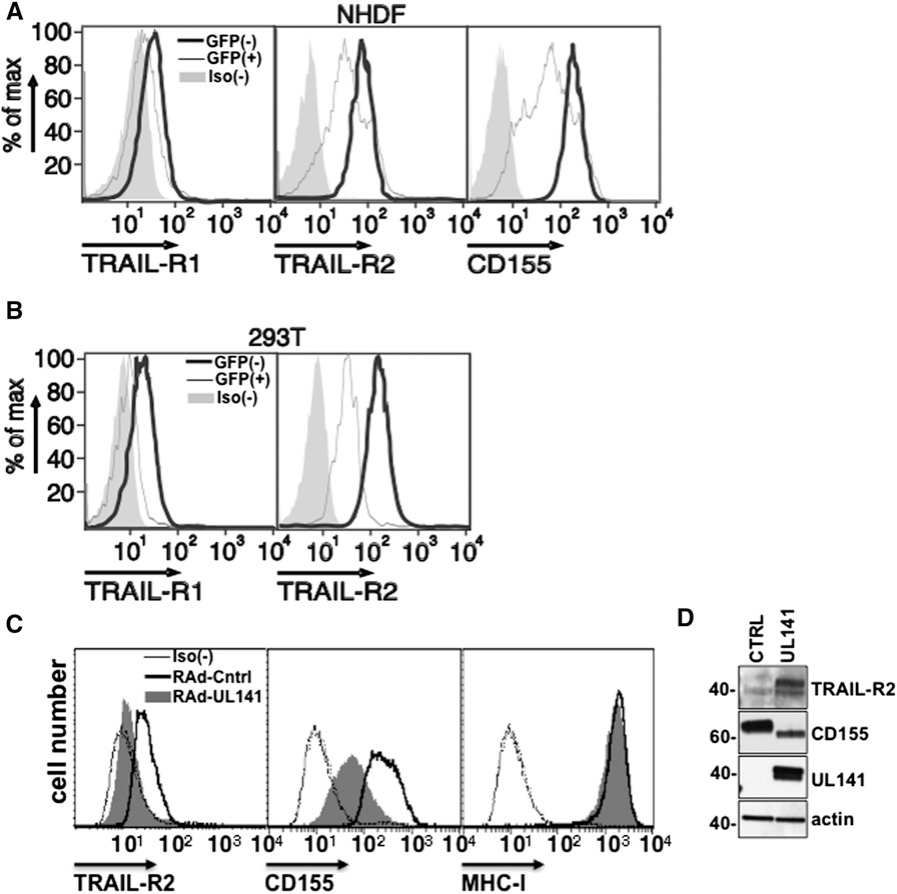
UL141 Is Sufficient to Inhibit Cell-Surface Expression of TRAIL DR (A) NHDF cells or (B) 293T cells cotransfected with UL141 and a GFP expressing plasmid were analyzed for cell-surface expression of the indicated proteins by flow cytometry 48 hr later. Black histogram, GFP(–) cells; gray histogram, GFP(+) cells; gray shaded histogram, isotype control binding to entire cell population. Human fibroblasts (HF-CAR) were infected (48 hr, MOI = 3) with RAd-CTRL or RAd-UL141 and analyzed for TRAIL-R2 expression by (C) flow cytometry and (D) Western blot. Similar results were obtained in three to five experiments.

Studies with HCMV deletion mutants demonstrated that UL141 was required to provide efficient downregulation of the TRAIL DRs at the cell surface. UL141 alone is sufficient to restrict CD155 cell-surface expression (Tomasec et al., 2005), but additional HCMV-encoded functions are needed to target CD112 (Prod’homme et al., 2010). We therefore sought to determine whether UL141 was able to target TRAIL DRs when expressed in isolation. A UL141 expression plasmid was transfected into both primary fibroblasts and 293T cells, and significant downregulation of TRAIL-R1 and TRAIL-R2 was observed, proving that UL141 alone is sufficient to suppress TRAIL DR expression (Figures 3A and 3B). Inhibition of TRAIL DRs was also observed when a UL141-GFP fusion protein was stably transfected into 293T cells, commensurate with enhanced accumulation of intracellular TRAIL-R2, as seen in HCMV-infected cells (data not shown). Additionally, an adenovirus vector encoding UL141 also restricted TRAIL DR levels on the cell surface and promoted its intracellular accumulation in both fibroblasts and epithelial cells (Figures 3C, 3D, and S2). Together, these experiments demonstrated that UL141 inhibits cell-surface expression and promotes intracellular accumulation of the TRAIL DR without the assistance of any additional HCMV-encoded function.

### UL141 Interacts Directly with the Human TRAIL Death Receptors

UL141 is a type I transmembrane glycoprotein with a short C-terminal cytoplasmic domain, and structural algorithms predict that it contains an immunoglobulin-like fold in its ectodomain (Tomasec et al., 2005). To determine whether UL141 targets the TRAIL DRs by directly binding to them, we expressed and purified the UL141 ectodomain (UL141ecto) as well as a fusion protein of the ectodomain with the Fc region of human IgG1 (UL141:Fc). The binding assay demonstrated an interaction between UL141:Fc and both TRAIL-R1:Fc and TRAIL-R2:Fc (Figure S3A). While UL141:Fc binds to the surface of human fibroblasts and 293T cells (data not shown), this result was not informative as CD155 is also expressed at high levels on most human cells. In contrast, UL141:Fc was incapable of binding to mouse NIH 3T3 fibroblasts (which express mTRAIL-R2), but transfection of hTRAIL-R2 into NIH 3T3 cells promoted strong binding of UL141:Fc (Figure S3B), formally showing that UL141 can interact with cell-surface-expressed hTRAIL-R2. Consistent with this result, binding between mouse TRAIL-R2:Fc and UL141:Fc was not observed in an ELISA-based assay (data not shown). Taken together, these data prove that UL141 binds directly to both human TRAIL DRs.

**Figure 4:**
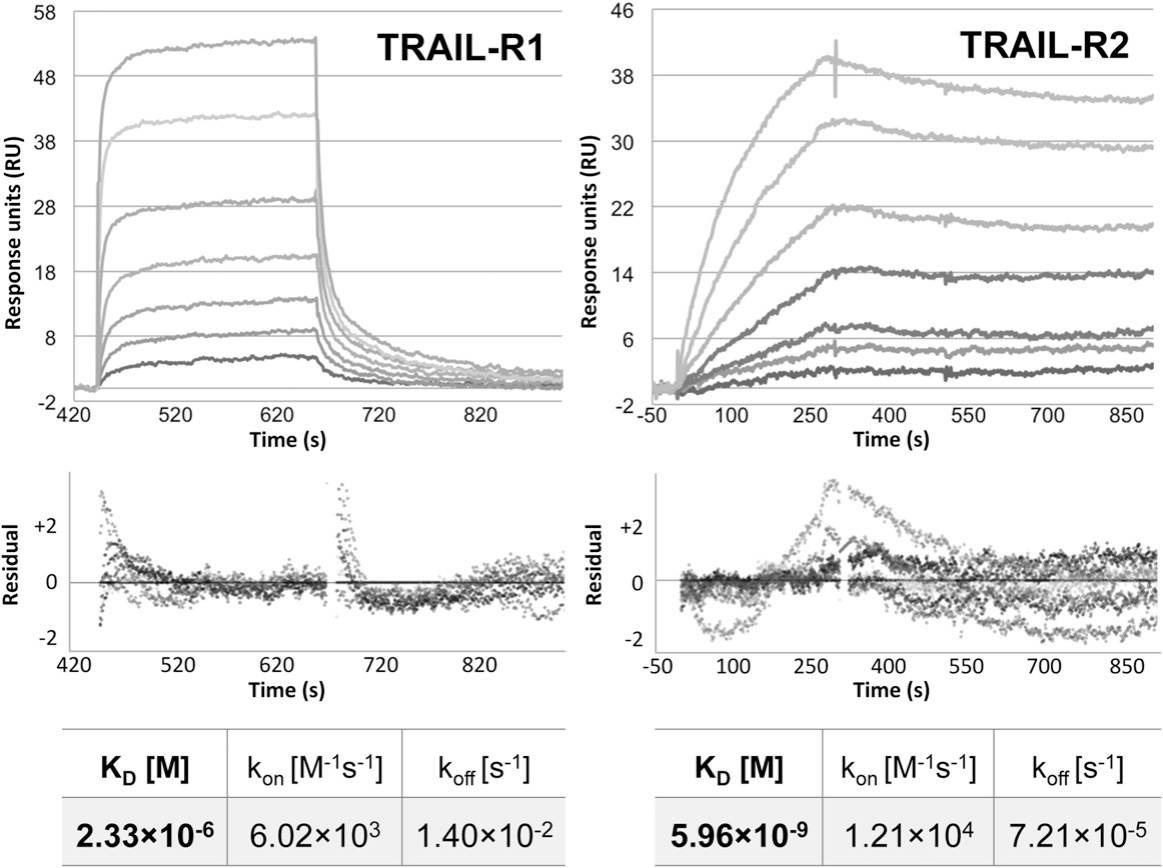
UL141 Binds Directly to the TRAIL DR Sensorgrams of UL141 binding to TRAIL-R1 (left) and -R2 (right). Each curve (top) represents the binding response of UL141 to both DR at a different concentration (0.78–50 μM, left; 0.016–1 μM, right). The corresponding residual statistics representing the deviation from the fitted data to the actual response values is shown below. The K_D_ of 2.3 μM and 6 nM were determined for UL141ecto binding to TRAIL-R1:Fc and TRAIL-R2:Fc, respectively, immobilized on the chip. See also Figure S3.

In order to determine the binding kinetics/affinity of UL141 for the TRAIL DRs, we conducted surface plasmon resonance analysis of UL141ecto binding to TRAIL-R1 and TRAIL-R2:Fc proteins (Figure 4). UL141 was found to bind to TRAIL-R2 with a K_D_ of 6nM, an affinity very close to that of TRAIL (2 nM [Truneh et al., 2000]). In contrast, UL141 bound to TRAIL-R1 with a dramatically lower affinity (K_D_ = 2.3 μM), with differences in both the association and dissociation kinetics being observed. The UL141 binding kinetics to TRAIL-R1 revealed only a 2-fold slower association rate (k_on_ = 6.0 × 10^3^ M^−1^ s^−1^), while dissociation was almost 200 times faster (k_off_ = 1.4 × 10^−2^ s^−1^), when compared to UL141 binding to TRAIL-R2-Fc (k_on_ = 1.2 × 10^4^ M^−1^ s^−1^, k_off_ = 7.2 × 10^−5^ s^−1^, Figure 4). Taken together, these results confirm direct binding of UL141 to both human TRAIL DRs, but with significantly lower affinity for TRAIL-R1, largely mimicking how TRAIL binds its two cognate DRs (Truneh et al., 2000).

The fact that UL141 interacts directly with the TRAIL DRs is interesting, as it shows no primary sequence or predicted structural homology to any TNF family ligands (Bodmer et al., 2002). This raised the possibility that UL141 might interact with additional members of the TNFR superfamily. To test this, we used UL141:Fc protein to stain 293T cells transfected with all the known TNFRs (Figure S3C) (Bossen et al., 2006). In this assay format, where positive controls were included for all receptor-ligand interactions, strong binding of UL141:Fc (∼5 μg/ml) was only detected to 293T cells transfected with TRAIL-R2. Notably, binding of UL141:Fc under these conditions was not detected to TRAIL-R1, most likely due to the relatively low binding affinity for this DR compared to TRAIL-R2, as seen in SPR analyses. Consequently, these data indicate that TRAIL-R2 appears to be the only member of the TNFR superfamily that is a specific, high-affinity target for UL141.

### Intracellular Retention of TRAIL-R2 in the Presence of UL141

**Figure 5:**
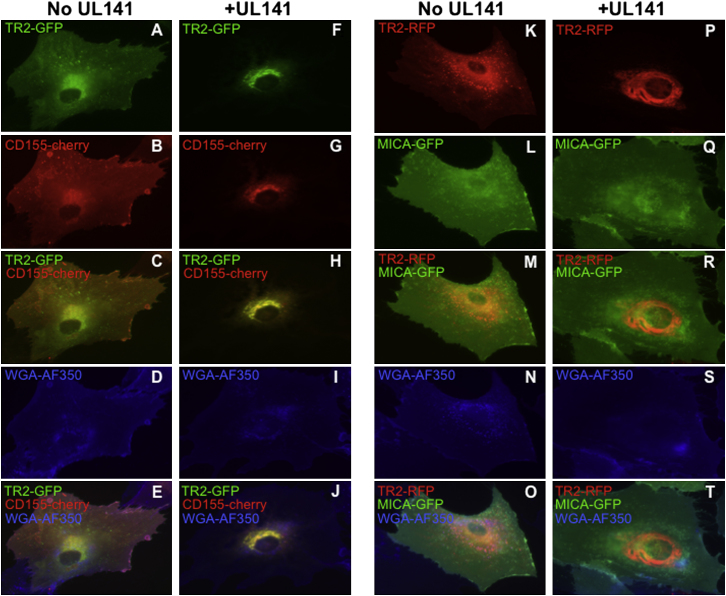
UL141 Restricts Expression of TRAIL DR to the Endoplasmic Reticulum Human fibroblasts were coinfected for 48 hr with adenovirus vectors expressing TRAIL-R2-ΔDeathDomain-GFP (TR2-GFP), TR2-RFP, CD155-cherry, or MICA-GFP, as indicated. A proportion of cells were also coinfected with adenovirus vector expressing UL141 (panels F–J and P–T). Slides were counter stained with WGA-AF350 to visualize the outline of the cells. See also Figure S4.

Expression of TRAIL DRs is largely localized to intracellular membrane compartments in lung and melanoma-derived cell lines, with a minority of the total protein present on the plasma membrane at steady state (Leithner et al., 2009; Zhang et al., 2000). Virtually nothing is known regarding the mechanisms that regulate the trafficking of TRAIL DRs through various cellular compartments, although ER stress has been shown to upregulate TRAIL-R2 cell surface levels and sensitize them to TRAIL-induced killing (Chen et al., 2007). To examine whether UL141 alters the subcellular localization of TRAIL-R2, we transduced fibroblasts with adenoviral recombinants (RAds) encoding UL141 and TRAIL-R2 constructs fused to C-terminal GFP or RFP tags and lacking an intact death domain (averting apoptosis mediated by overexpression of full-length TRAIL-R2) (e.g., RAd-TRAILR2.GFP) (Figure 5). For comparison, cells were also transduced with RAd-CD155.RFP or RAd-MICA.GFP. TRAILR2.GFP and TRAILR2.RFP were expressed throughout a variety of intracellular membrane compartments, on the cell surface, and colocalized with endosomal markers (Figures 5A and 5K and data not shown). In contrast, when this DR and UL141 were coexpressed, TRAIL-R2 was restricted in large part to the ER, (Figures 5F, 5P and S4A). UL141 also localizes primarily to the ER, and not to the *trans* medial Golgi complex, in the absence of coexpressed TRAIL-R2 or CD155 (Figure S4B). This pattern of intracellular compartmentalization was similar to that observed in cells transduced with RAd-CD155.RFP and UL141 (Figures 5G and 5H). The interaction between TRAIL-R2 and UL141 was specific, as UL141 did not alter trafficking/localization of MICA.GFP (Figures 5P and 5Q), which is known to be downregulated from the cell surface through the action of HCMV UL142 (Ashiru et al., 2009; Chalupny et al., 2006). Importantly, similar localization of TRAIL-R2 to the ER was seen in cells infected with wild-type Merlin but not MerΔUL141 (Figure S4C). Taken together, these data support biochemical analyses showing that UL141 redirects and/or restricts TRAIL DR expression to an intracellular membrane compartment(s), mainly the endoplasmic reticulum. Interestingly, this differs from the mechanism used by adenovirus E3 region proteins, which target TRAIL DRs for lysosomal degradation (Benedict et al., 2001).

### UL141 Functions Nonredundantly to Restrict TRAIL-Mediated Killing

**Figure 6:**
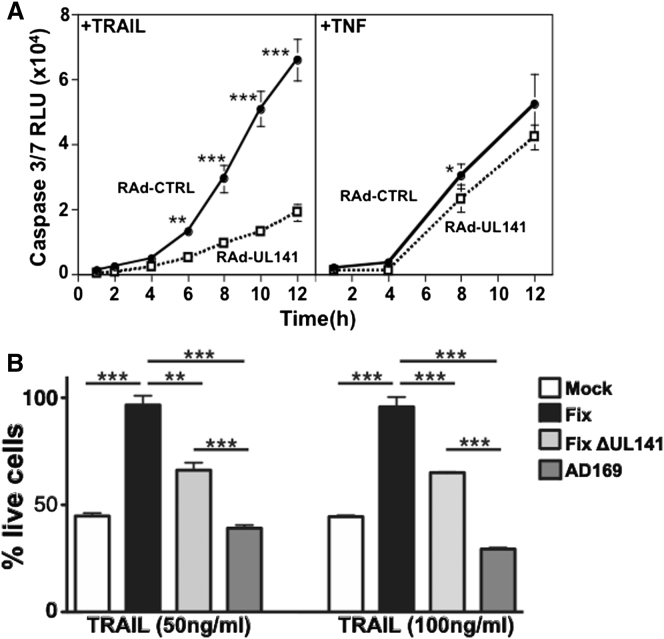
UL141 Inhibits TRAIL-Mediated Apoptosis (A) Human fibroblasts (HF-CAR) were infected with RAd-UL141 or RAd-CTRL (48 hr, MOI = 3), incubated with TRAIL or TNFα as indicated, and analyzed for caspase-3/caspase-7 activation (n = 4, error bars represent the SD). (B) NHDF cells were either mock infected or infected with the indicated HCMV viruses at an MOI of ∼2. Forty-eight hours later, 50 or 100 ng/ml purified hTRAIL plus 5 μg/ml cycloheximide (CHX) was added for an additional 48 hr before assessment of cell viability. In all cases, the percentage of live cells was calculated by normalization of TRAIL+CHX-treated cells to cultures treated with CHX only. In (A), a Student’s t test was used for statistical analysis, and the 8 hr time point in TNFα-treated cells has a p value of 0.048. In (B), statistical analysis was performed with the one-way ANOVA (for both groups, ^∗∗∗^p < 0.0001) and displayed are Tukey’s multiple comparison post test results. Error bars in (B) represent mean ± SEM.

We sought to investigate whether the intracellular sequestration of TRAIL-R2 by UL141 desensitized cells to TRAIL-mediated apoptosis. To test this, we treated human fibroblasts transduced with UL141 with soluble TRAIL (Figure 6A). UL141-expressing cells showed dramatically reduced activation of caspase-3/caspase-7, proving that UL141 can desensitize cells to apoptotic signaling mediated by the TRAIL DR. This effect was specific, as the sensitivity of UL141-expressing cells to TNF-mediated apoptotic signaling was not overtly altered (Figure 6A).

Next, the effect that UL141 restriction of TRAIL DR cell-surface expression had on altering the sensitivity of HCMV infected cells to TRAIL killing was analyzed (Figure 6B). Fibroblasts infected with FIX were completely protected from TRAIL-mediated killing. In contrast, FIXΔUL141-infected cells were significantly more sensitive to TRAIL-induced apoptosis, which was notable, as other potentially redundant mechanisms targeting DR signaling are still operable in this mutant virus (e.g., UL36-mediated inhibition of caspase-8 activation [Skaletskaya et al., 2001a]). To further explore this issue, we analyzed the sensitivity of cells infected with HCMV strain AD169 to TRAIL killing, as this strain encodes a nonfunctional UL36 (Skaletskaya et al., 2001a) in addition to lacking UL141. AD169-infected fibroblasts were even more sensitive to TRAIL killing than those infected with FIXΔUL141, consistent with a model in which both UL36 and UL141 are likely to contribute to the inhibition of TRAIL DR signaling. Taken together, these studies demonstrate that UL141 restriction of TRAIL DR cell-surface expression provides nonredundant protection against TRAIL-mediated apoptosis in HCMV-infected cells.

### UL141 Inhibition of TRAIL DRs Contributes to NK Cell Inhibition

**Figure 7:**
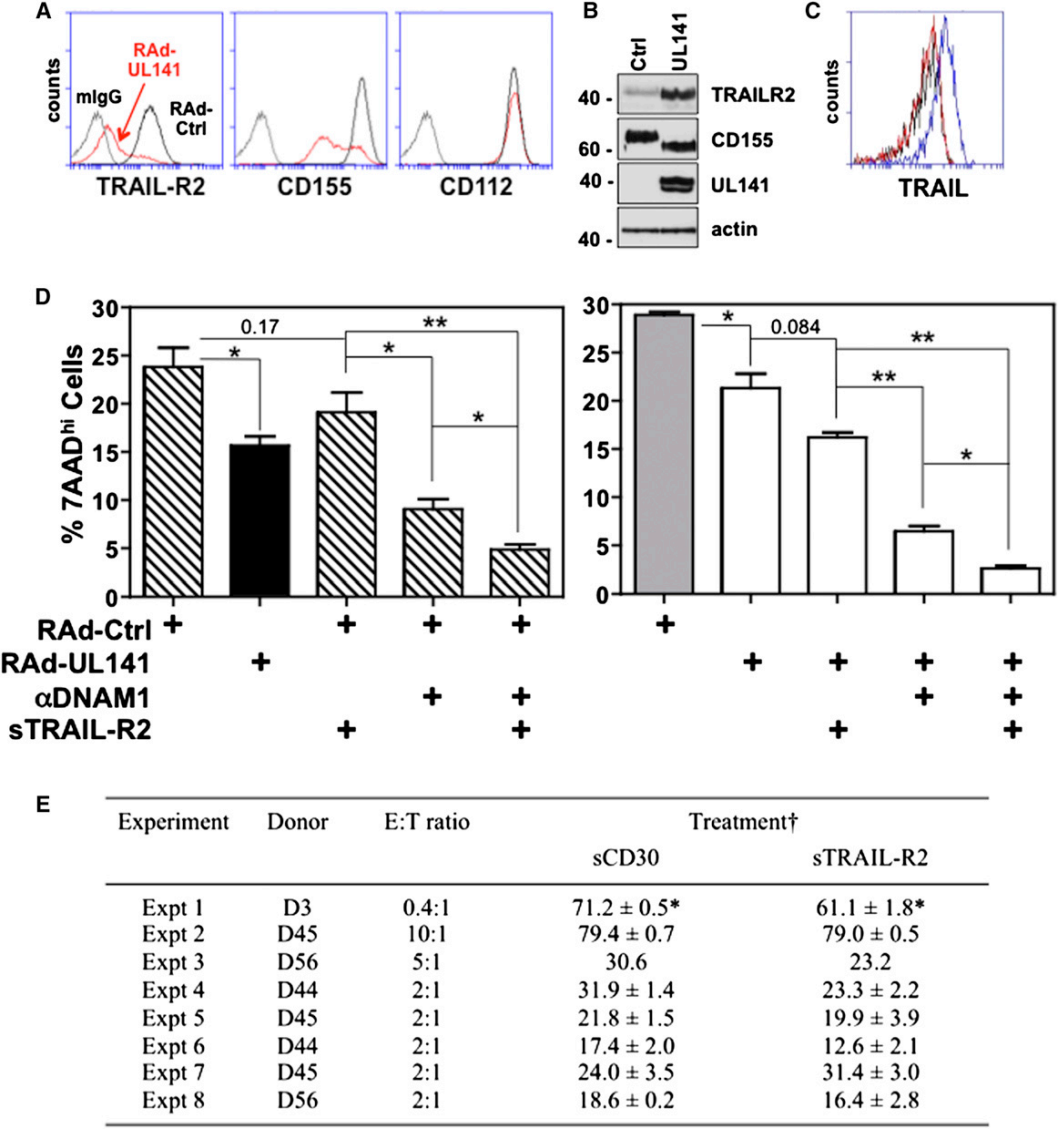
UL141 Blockade of Both TRAIL DR and CD155 Contributes to NK Cell Inhibition (A) RAd control or UL141 transduced A549 cells were analyzed for expression of TRAIL-R2, CD155 and CD112 at the time of NK cell addition by flow cytometry. (B) Western blot of RAd-transduced A549 cells. (C) Expression of TRAIL by IFNα activated (blue) or unactivated (red) human NK cells assessed by flow cytometry. (D) IFNα-activated NK cells were purified from human peripheral blood and added to A549 lung epithelial cells transduced with either control adenovirus vector (Rad-Cntrl) or Rad-UL141 (effector to target [E:T] ratio of 2). Blocking αDNAM-1 antibody or blocking soluble TRAIL-R2 (10 μg/ml) was added to cultures where indicated (+), and control mIgG or sCD30 was added as a control to the other cultures. Apoptosis of A549 cells was assessed 4 hr later. Shown are two representative experiments of more than six performed. (E) Summary of NK killing data from eight separate experiments and four different donors. †Data are mean ± SEM from n = 1 to 3 wells. ^∗^Bonferroni post test shows significance at p < 0.05. See also Figure S5.

Lung epithelial cells expressing UL141 exhibited markedly reduced cell-surface expression of TRAIL-R2 and CD155, while intracellular levels of both molecules increased (Figures 7A and B). Our previous studies revealed UL141 to be a potent inhibitor of NK cell killing via downregulation of the DNAM-1-activating ligands CD155 and CD112 (Tomasec et al., 2005) but were not designed to measure contributions of NK-cell-mediated apoptosis regulated by DR signaling. TRAIL is poorly expressed in the majority of human NK cells isolated directly from peripheral blood, although, interestingly, it is present at high levels in the small percentage of CD56^hi^ NK (Figure S5). Consequently, “bulk” NK cells were first activated with interferon α (IFNα) (Figure 7C), a physiologically relevant inducer of TRAIL during viral infection in vivo (Sato et al., 2001; Takeda et al., 2001). Cellular targets transduced with control adenovirus vector were significantly more sensitive to apoptosis mediated by these NK effectors than those expressing UL141 (Figure 7D). Anti-DNAM-1 blocking antibody reduced NK cell killing by ∼65%, and a similar reduction was seen in both control cells and those expressing UL141. Blocking TRAIL-mediated effector functions with soluble TRAIL-R2 also trended toward reducing NK killing (∼30% reduction, p = 0.08), and analyzing results of eight separate experiments from four individual NK cell donors revealed that inhibiting TRAIL does significantly reduce NK-cell-mediated killing of UL141-expressing cells (p = 0.0081, two-way ANOVA, Figure 7E). Consistent with an important contribution of TRAIL in NK-cell-mediated killing, the addition of soluble TRAIL-R2 in combination with anti-DNAM-1 reduced killing to an even greater extent, with more-robust reductions seen in UL141-expressing targets when compared to control cells (∼11-fold versus ∼4.5-fold). These results highlight a selective importance of UL141 in promoting resistance to TRAIL. Notably, the observed sensitivity of UL141-expressing targets to NK cell killing via TRAIL and DNAM-1 is mediated by “residual” levels of CD155 and TRAIL-R2 in these target cells (Figure 7A) and is very likely relevant given that incomplete inhibition of their cell-surface expression is also seen in HCMV-infected cells (see Figures 1 and 2).

## Discussion

Here we uncover a herpesvirus protein that acts to inhibit TRAIL-mediated apoptosis by specifically targeting cell-surface expression of the TRAIL DRs. HCMV now joins a growing list of DNA and RNA viruses that modulate signaling by the TRAIL/TRAIL-R cytokine system. This study highlights the fundamental role that signaling by TNF family cytokines plays in driving the evolution of host attack and viral retort that is critical for the success of persistent viral pathogens. Our data are consistent with a model in which gpUL141 binds directly to the ectodomain of the human TRAIL DRs in the lumen of the ER, sequestering them as a stable complex as both proteins accumulate. Consequently, transport through the Golgi apparatus and onward is impeded, and cells are desensitized to extrinsically mediated TRAIL killing. Notably, HCMV infection had previously been reported to sensitize cells to TRAIL and induce DR expression (Sedger et al., 1999), but this can now be explained in those studies by the use of the high-passage AD169 strain, which encodes a defective UL36 and also lacks the entire UL/*b*′ genomic region (Skaletskaya et al., 2001a; Cha et al., 1996). TRAIL expression is upregulated on the surface of HCMV-infected DCs, promoting the death of virus-specific T cells that encounter them (Raftery et al., 2001). Perhaps the commensurate restriction of the TRAIL DRs by UL141 is necessary to protect infected DCs from TRAIL-mediated fratricide and/or suicide. TRAIL mRNA is also highly induced by HCMV in placental fibroblasts via the action of type I IFN (Andrews et al., 2007), suggesting a similar mechanism to thwart host immunity may operate during congenital infection (Nigro and Adler, 2011). Consequently, HCMV may utilize the immune-suppressive activities of TRAIL to its advantage, while simultaneously inhibiting its action in infected cells via the action of UL141. Intriguingly, and indicative of a multifaceted role for the TRAIL DRs in CMV defense, myeloid cells from TRAIL-R2^−/−^ mice produce increased levels of inflammatory cytokines when infected with mouse CMV (MCMV), promoting increased NK cell activation and enhanced control of viral replication in the spleen, but not the liver (Diehl et al., 2004). Although the mechanism(s) for this inhibitory role of TRAIL DR signaling in mice is not currently understood, it illustrates the importance of considering cell-type- and tissue-specific roles for the TRAIL cytokine system in regulating antiviral immune defenses.

Although CD155 and CD112 share homology, as do TRAIL-R1 and TRAIL-R2, these proteins show no primary sequence or predicted structural homology to each other. The crystal structure of UL141 in complex with TRAIL-R2 reveals that its Ig domain is utilized to interact with TRAIL-R2 (C.A.B. and D.M.Z., unpublished data), but whether a similar mechanism is used to bind CD155 remains an open question. Until quite recently, interacting partners for the TNFRs were thought to be restricted to the trimeric TNF family ligands. However, when HVEM/TNFRSF14 was found to bind the inhibitory cosignaling receptor BTLA, this dogma was reassessed (Sedy et al., 2005). Interestingly, HCMV UL144 also targets this signaling system. UL144 is a partial functional ortholog of HVEM that binds to BTLA, but not to LIGHT, and potently inhibits T cell proliferation (Cheung et al., 2005; Sedý et al., 2008). Our data highlight UL141 as a non-TNF family protein that can interact with the ectodomain of the TRAIL DRs, providing further evidence for TNFR binding partners that extend outside of the canonical family. It is intriguing that UL141 binds to TRAIL-R1 with a much lower affinity than to TRAIL-R2, as this mimics what is seen for TRAIL binding (Truneh et al., 2000) and suggests a biological significance that is currently underappreciated. Along these lines, TRAIL-R2 is normally highly expressed intracellularly in uninfected cells, with UL141 greatly enhancing these levels. The UL141-mediated accumulation of TRAIL DR, as well as CD155, raises the intriguing possibility that these host-cell proteins may possess yet-to-be described roles as intracellular signalers.

UL141 is now known to be required for restricting the cell-surface expression of at least four cellular proteins: TRAIL-R1, TRAIL-R2, CD155, and CD112. CD155 was the first identified target of UL141 (Tomasec et al., 2005), and the decreased sensitivity of cells expressing UL141 to NK cell killing is ascribed in part to its inhibition of NK cell activation via DNAM1. DNAM1 is an activating receptor that is a key initial component of NK cell activation/licensing. Killing itself can then be mediated through cytotoxic granule release in conjunction with signaling by TNF family ligands that bind to cognate death receptors. In order to assess whether UL141 restriction of TRAIL DR expression contributes to NK inhibition, we developed a physiologically relevant assay in which IFNα-activated NK cells were used as effectors. Interestingly, although CD56^hi^ NK cells only compose a small percentage of circulating NK cells in peripheral blood (∼5%), these cells express high levels of TRAIL (Figure S5). Consequently, since many more NK cells present in human tissues are CD56^hi^ (Poli et al., 2009), this suggests that our in vitro assays with NK cells isolated from blood might underrepresent the contribution of TRAIL in NK cell control of HCMV. Also, it is important to distinguish the distinct roles played by DNAM-1 and TRAIL in NK cell killing. DNAM-1 is involved in the 1° “decision-making” process, while TRAIL participates in executing that decision. This is highlighted by the fact that in our assays, antibody blockade of DNAM-1 would not affect the negative signal mediated by CD155 binding to its “paired” NK cell inhibitory receptor, TIGIT (Yu et al., 2009). In total, UL141 imposes a multilayered strategy to inhibit NK cells through dampening both their initial activation and downstream killing.

Finally, targeting of TRAIL DRs, TNFR-1, and HVEM by the UL138-144 UL/*b*′ locus now defines this gene cluster as being highly focused on modulating signaling by the TNFR superfamily. Additionally, UL141 and UL142 (Ashiru et al., 2009) stand out within this cluster as having proven NK-cell-modulating functions. As NK cells also express BTLA, perhaps UL144 will soon join their ranks. Notably, UL141 and UL144 are also conserved and immediately adjacent ORFs in the rhesus CMV genome (Hansen et al., 2003), further emphasizing their likely importance in CMV modulation of primate innate immunity.

## Experimental Procedures

### Cells and Virus

NHDFs were obtained from Clonetics (San Diego, CA), immortalized HFFs are described (McSharry et al., 2001), and 293T cells were from the ATCC (CRL-11268). All cells were cultured in Dulbecco’s modified Eagle’s medium (DMEM) supplemented with 10% fetal bovine serum, Pen/Strep, and L-glutamine (GIBCO). Insulin and bFGF (Sigma-Aldrich) were added to NHDF media. Cells were verified to be mycoplasma negative. AD169 was acquired from the ATCC (VR-538, used p2-5), and Toledo was a kind gift from S. Starr (Philadelphia, used p12-15). Mutagenesis of FIX was performed as described (Hahn et al., 2002), and additional primer sequences are in supplemental Table S1. MerΔUL141 generation was described (Prod’homme et al., 2010). HCMV virus was generated by BAC transfection into fibroblasts as described (Hahn et al., 2002; Stanton et al., 2010).

### RNA Isolation and Analysis

Total RNA was isolated from HCMV infected cells with TRIzol (Roche) followed by an RNeasy mini kit (QIAGEN, Hilden, Germany). Complementary DNA generation and real-time quantitative PCR analysis was as described (Schneider et al., 2008), and primer sequences can be requested. For RACE analysis, a 5′/3′ RACE kit was used (Roche), and primers were as follows: UL141-5′, 5′-CCGGCGACGTGGTCTCATAA-3′; UL141-3′, 5′-ATCGCGGCATTTTTGGGATT-3′. The amplified products were purified by agarose gel and sequenced.

### Flow Cytometry

HCMV-FIX-infected 6-well dishes of NDHFs or HFFs were detached with diluted trypsin, washed in PBS, and resuspended in PBS + 2% fetal calf serum. Cells were incubated with 1° antibody for 20–30 min on ice, followed by anti-mouse IgG1 biotin (BD) and Streptavidin-APC (PharMingen) if needed, and fixed with 1% paraformaldehyde. Anti-TRAIL-R1 and anti-TRAIL-R2 (HS101 and HS201, Alexis), anti-MHCI (W6/32, eBioscience), and anti CD155-PE (Biolegend) used at 5 μg/ml. Samples were acquired with a BD LSRII or FACScalibur flow cytometer and analyzed with FlowJo software (Tree Star). For Merlin infections, essentially the same methods were used, with secondary detection using anti-mouseAF647 (Molecular Probes, A-21238). Data were analyzed with Accuri/CFlowPlus. UL141 transfected 293T cells and NHDFs were analyzed similarly, as were adenovirus-transduced HFFs and purified human NK cells.

### Cell Death Assays

MTT cell viability assays in HCMV-infected NHDFs (Benedict et al., 2001) and caspase-3/caspase-7 activation assays (Skaletskaya et al., 2001a) were performed essentially as described. See the Supplemental Experimental Procedures for details.

### Plasmids, Adenovirus, Proteins, and Transfections

Plasmid vectors for expressing Fc-fusion proteins are described (PCR3-Fc) (Schneider, 2000), with details available in the Supplemental Experimental Procedures. Adenovirus vectors expressing UL141 are described (Tomasec et al., 2005). Generation of TRAILR2ΔDD.GFP, TRAILR2ΔDD.RFP, CD155.RFP, and MICA.GFP recombinant adenoviruses is described (Stanton et al., 2010), with modifications available in the Supplemental Experimental Procedures. Fc-fusion proteins used in ELISA and SPR were purified by protein A affinity from transfected 293T cell supernatants, except for TRAIL-R1:Fc (R&D Systems). For SPR studies, cell supernatants from SF9 cells transduced with baculovirus expressing His-tagged UL141ecto were collected after 3 days at 27.5°C (MOI = 3) and were purified with Ni^2+^-affinity chromatography followed by cation-exchange chromatography with MonoS (GE Healthcare) and gel filtration (Superdex S200, GE Healthcare) by fast protein liquid chromatography.

Dishes (6-well) of 293T were transfected with 2 μg UL141 plasmid as described (Cheung et al., 2005). UL141 plasmid (1 μg) was cotransfected into NHDFs with 0.5 μg provided control GFP plasmid according to manufacturer’s instructions (AMAXA).

### Surface Plasmon Resonance Studies

SF9 cell purified UL141 was exchanged to Biacore running buffer, TRAIL DR Fc fusion proteins and hLTβR:Fc (negative control) were immobilized on an on an anti-human Fc capture chip, and binding was analyzed with a Biacore 3000 (GE Healthcare) essentially as described (Wang et al., 2010). See the Supplemental Experimental Procedures for more details.

### Western Blots

Cells were dissolved in NuPAGE LDS sample buffer (Invitrogen), and proteins were resolved on NuPAGE Novex 10% Bis-Tris gels (Invitrogen), transferred to nitrocellulose membrane (Hybond-C, GE), and treated with Antibody Extender reagent (Pierce) per themanufacturers’ instructions. Antibodies used to probe membranes were as follows: TRAILR2 (R&D, AF631), CD155 (5D1) (Aoki, JBC, 1994:8431), UL141 (Tomasec et al., 2005), and actin (Sigma A-2066). Secondary antibodies were anti-mouse-HRP (BioRad 170-6516), anti-rabbit-HRP (BioRad 170-6515), and anti-goat-HRP (SantaCruz sc-2056).

### Immunofluorescence

Human fibroblasts (NPi) (Tomasec et al., 2000) were coinfected with the relevant adenovirus vectors, and 48 hr after infection cells were fixed with 4% paraformaldehyde, stained with WGA-AF350 (Molecular Probes W11263), and imaged on Leica DMIRBE microscope with Improvision Openlab software.

### NK Cell Killing Assays

Purification of “bulk” NK cells from IFNα-activated human peripheral blood mononuclear cell cultures has been described (Tomasec et al., 2005). See the Supplemental Experimental Procedures for more details.

### Statistical analysis

Unless otherwise noted, statistical significance was analyzed by the Student’s t test, and data represent the mean ± SEM.

## Supplemental Information

Document S1. Figures S1–S5, Table S1, and Supplemental Experimental Procedures
