# Supplementary material for: Human Cytomegalovirus Glycoprotein UL141 Targets the TRAIL Death Receptors to Thwart Host Innate Antiviral Defenses
Source: Cell Host Microbe. 2013 Mar 13;13(3):324–35. doi: 10.1016/j.chom.2013.02.003 (PMC3601332; doi:10.1016/j.chom.2013.02.003)
Supplement: Document S1. Figures S1–S5, Table S1, and Supplemental Experimental Procedures [file mmc1.pdf]

Cell Host & Microbe, Volume *13*

Supplemental Information

Human Cytomegalovirus Glycoprotein UL141 Targets the TRAIL Death Receptors to Thwart Host Innate Antiviral Defenses

Wendell Smith, Peter Tomasec, Rebecca Aicheler, Andrea Loewendorf, Ivana Nemčovičová, Eddie CY Wang, Richard J. Stanton, Matt Macauley, Paula Norris, Laure Willen, Eva Ruckova, Akio Nomoto, Pascal Schneider, Gabriele Hahn, Dirk M. Zajonc, Carl F. Ware, Gavin W.G. Wilkinson, and Chris A. Benedict

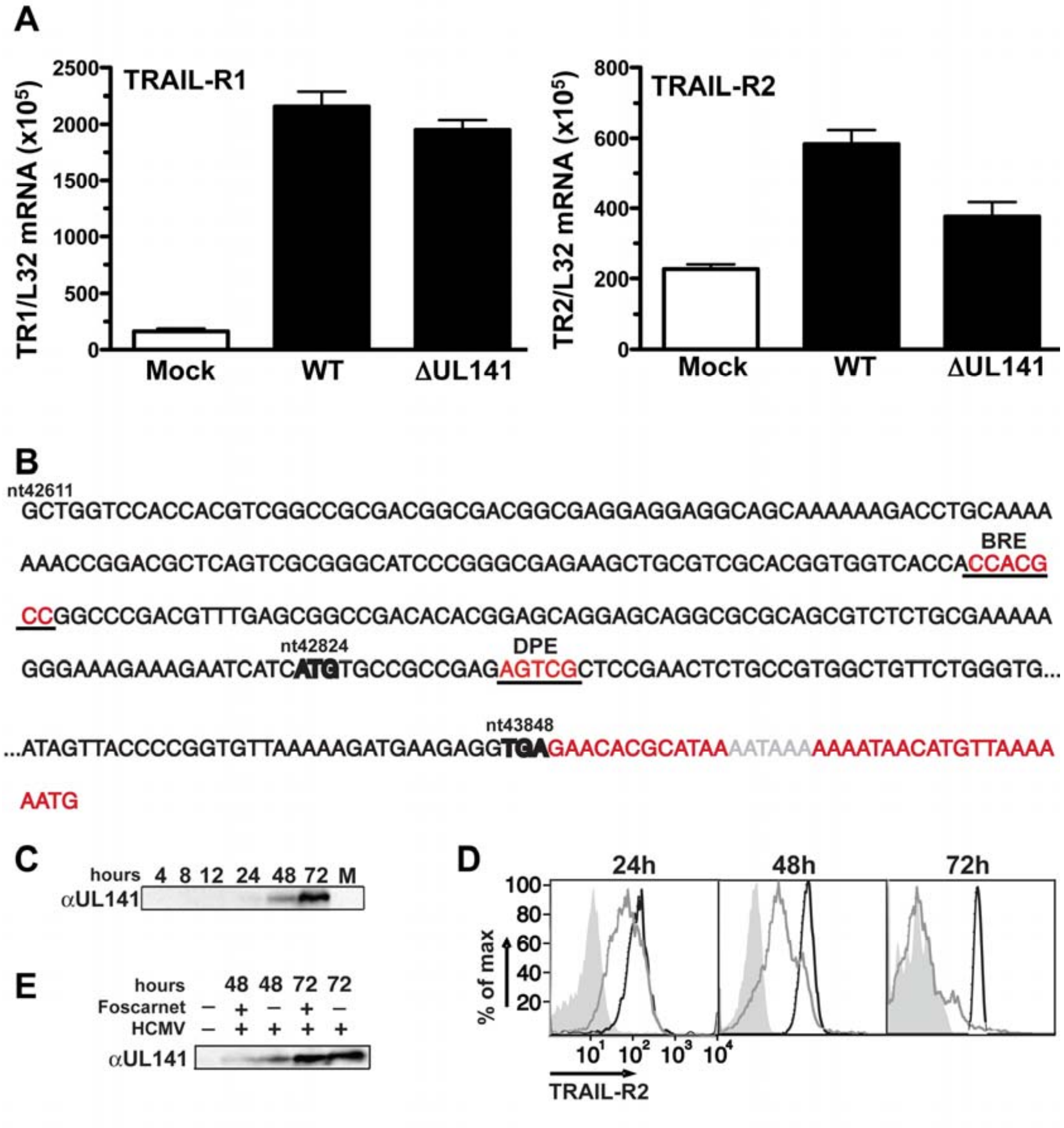

Figure S1. HCMV UL141 expression and affect on TRAIL DR mRNA expression, Related to Figure 1. A) NHDF cells were mock, FIX wild-type (WT) or FIXΔUL141 infected at an MOI of ~2. Cells were harvested 72 hours after infection for isolation of total cell RNA and subsequent RT-qPCR analysis of TRAIL-R1 or –R2 mRNA levels. Primers used were: TR1(+) 5'-TGGGT CAACAAAACTGGACG-3', TR1(-) 5'-CTTCCATCCTCTCCAAGGCAT-3'; TR2(+) 5'-CCAAGACCCTTGTGCTCGTT-3', TR2(-) 5' GTGATCAGAGCAGACTCAG CTGA-3'. Shown is the average of 3 separate infected wells +/- SD, and DR mRNA values are normalized to the expression of L32 mRNA ($x10^5$). B) The *ul141* mRNA transcript sequence as determined by RACE analysis of HCMV FIX infected cells (MOI ~ 2). Bold sequence, start and stop codon; underlined red text, TFIIB recognition element (BRE) and DPE (potential downstream promoter element, no obvious TATA box present); light grey, polyadenylation signal. 3' UTR shown in red. Nucleotide numbers correspond to the published FIX BAC sequence (Genbank accession #AC146907). C) NHDF were infected, harvested at the indicated hours(h) post infection and analyzed by SDS-PAGE followed by Western blot to detect UL141. D) NHDF cells were infected with HCMV FIX WT at an MOI of 0.5 and were analyzed by flow cytometry with antibodies specific for HCMV UL44 and TRAIL-R2. Black histogram, UL44- cells; grey histogram, UL44+ cells in culture; shaded histogram, isotype control E) NHDF were infected with FIX WT (MOI ~2)+/- Foscarnet treatment (250μg/ml) for an additional 48 or 72 hours. Cells were harvested and HCMV UL141 protein was detected by SDS-PAGE followed by Western blot. M, mock.

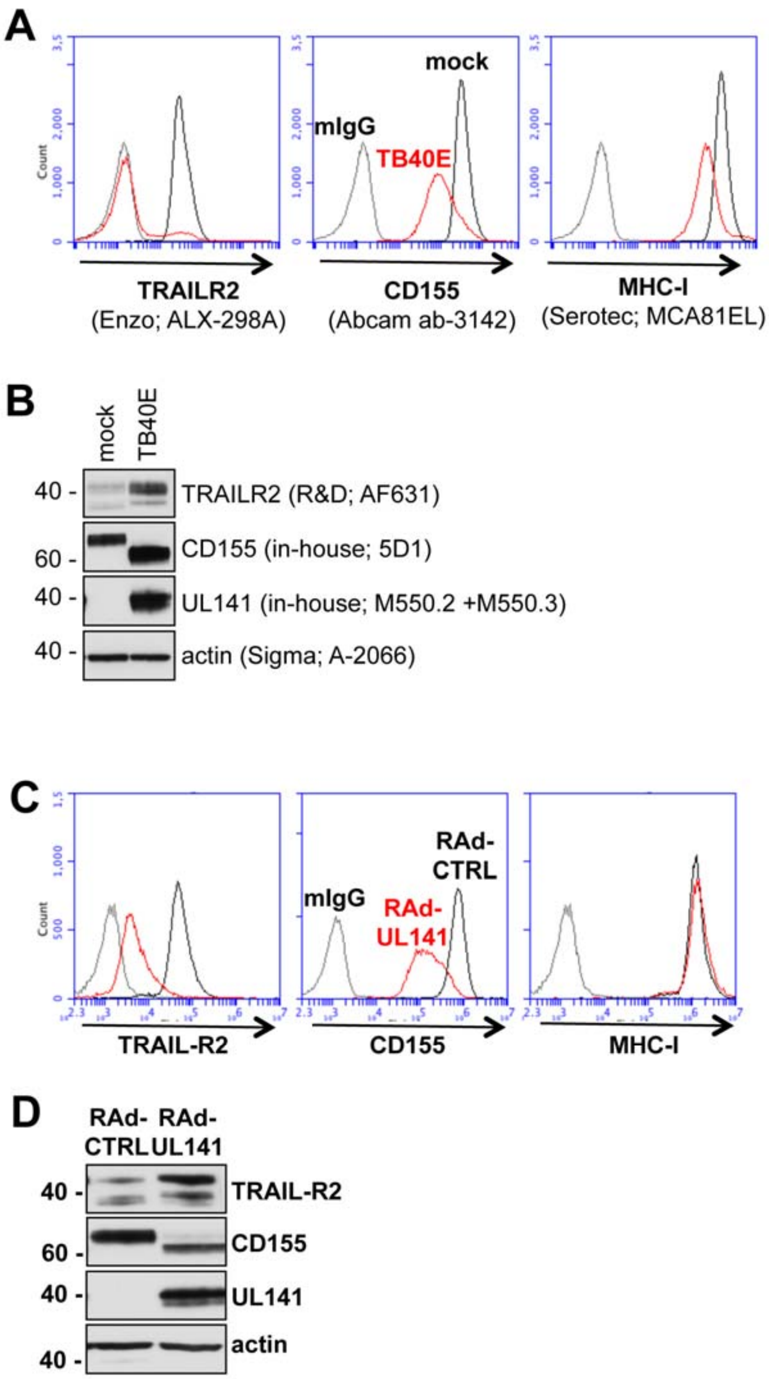

Figure S2. HCMV UL141 restricts cell surface expression of TRAIL-R2 in epithelial cells, Related to Figure 2. In A and B, ARPE-19 cells were infected with HCMV TB40E strain (MOI~100, based on pfu values calculated in fibroblasts). 72h after infection, cells were analyzed by (A) flow cytometry and (B) western blot. In C and D, ARPE-19 cells were infected with adenovirus vector expressing UL141 (RAd-UL141) or control adenovirus (RAd-CTRL) at MOI=20. 72 hours later cells were analyzed by flow cytometry (C) and western blot (D). Companies and catalog numbers for antibodies used are indicated.

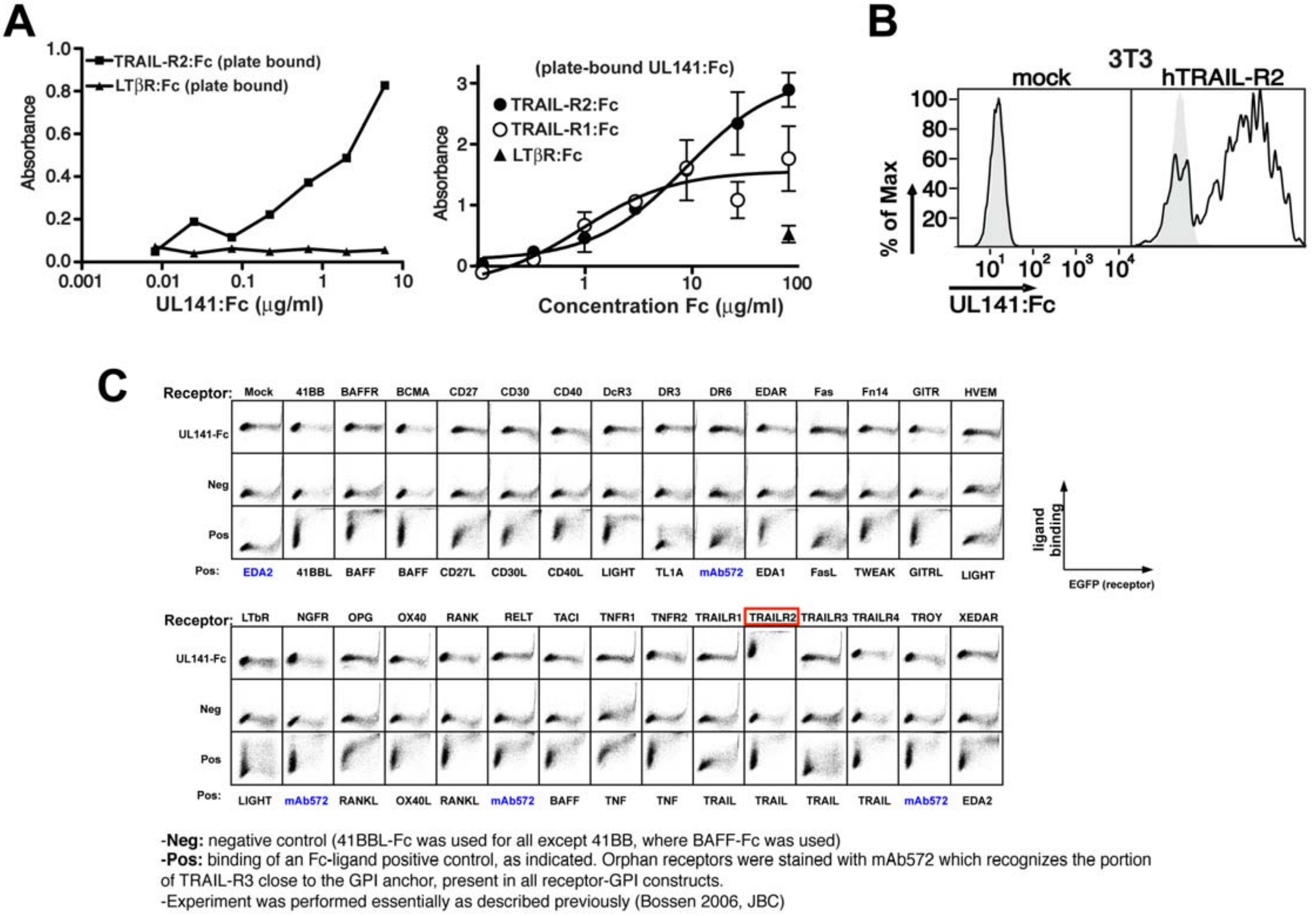

Figure S3. UL141 binds directly to the TRAIL DRs, but no other TNFRs, Related to Figure 4. A) ELISA plates were coated with either TRAIL-R2:Fc or mLTβR:Fc (negative control), and UL141:Fc protein was added at increasing concentration. Binding of UL141:Fc was detected using a UL141-specific monoclonal antibody. B) ELISA plates were coated with UL141:Fc and either TRAIL-R2:Fc, TRAIL-R1:Fc were added to plates at increasing concentrations, with or mLTβR:Fc added as a negative control. Fc protein binding was detected using DR-specific monoclonal antibodies. Shown are the mean of 3 duplicate wells +/- SEM B) 3T3 mouse fibroblasts were either mock transfected (left histogram) or transfected with a hTRAIL-R2 GPI-linked expression plasmid (right histogram) and stained with UL141:Fc (black) or hIgG (gray shaded). C) 293T cells were transfected with various TNFR expression plasmids in combination with a GFP expression plasmid, followed by incubation with either UL141:Fc, 4-1BBL:Fc (negative control) or the appropriate TNF-ligand:Fc protein (positive control). Experiment was performed essentially as previously described (Bossen et al., 2006).

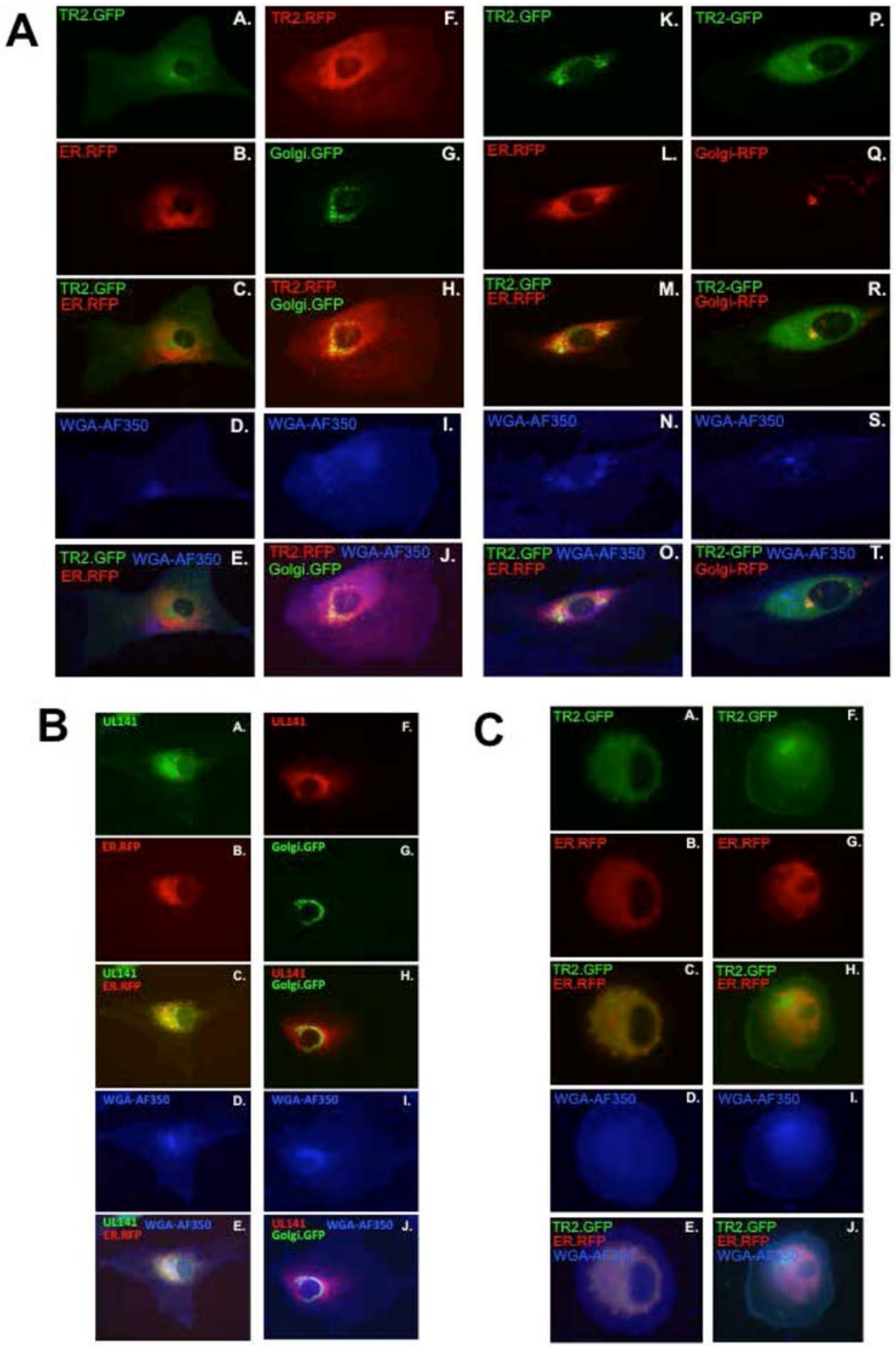

Figure S4. UL141 and TRAIL DR subcellular localization in transfected and infected cells, Related to Figure 5. *Panel A*: NPi cells were transduced with RAd vectors expressing TRAILR2ΔDD-GFP (TR2.GFP; panels A-E and K-T), TRAILR2ΔDD-RFP (TR2.RFP; panels F-J), UL141 (panels K-T), ER marker-RFP (ER.RFP; panels A-E and K-O) or trans-medial Golgi marker-GFP (Golgi-GFP;

panels F-J and P-T) as indicated. 48h later the cells were fixed and stained with WGA-AF350 where indicated. In the presence of UL141, TRAIL-R2 localized predominantly within the ER (panel M.). TRAIL-R2 alone partially colocalized to the ER (panel M) and occasionally/atypically to the Golgi (panel R.). *Panel B:* NPi cells were co-infected for 48h with adenovirus vectors expressing UL141 (all panels) and either an RFP-tagged ER marker (ER.RFP; panels A-E) or GFP-tagged trans-medial Golgi marker (Golgi.GFP; panels F-J). Prior to imaging, cells were fixed with paraformaldehyde and stained with anti-UL141 antibody and/or wheat germ agglutinin (WGA-AF350, marks cellular outline). *Panel C*: NPi cells were infected with HCMV Merlin (panels A-E) or HCMV MerlinΔUL141 (panels F-J). The following day, cells were transduced with adenovirus vectors expressing TRAIL-R2ΔDD-GFP (TR2.GFP), and ER marker-RFP (ER.RFP). The next day, cells were fixed, stained where indicated with WGA-AF350 and analyzed by fluorescence microscopy. Construction of ER and Golgi marker plasmids is described in the supplemental methods.

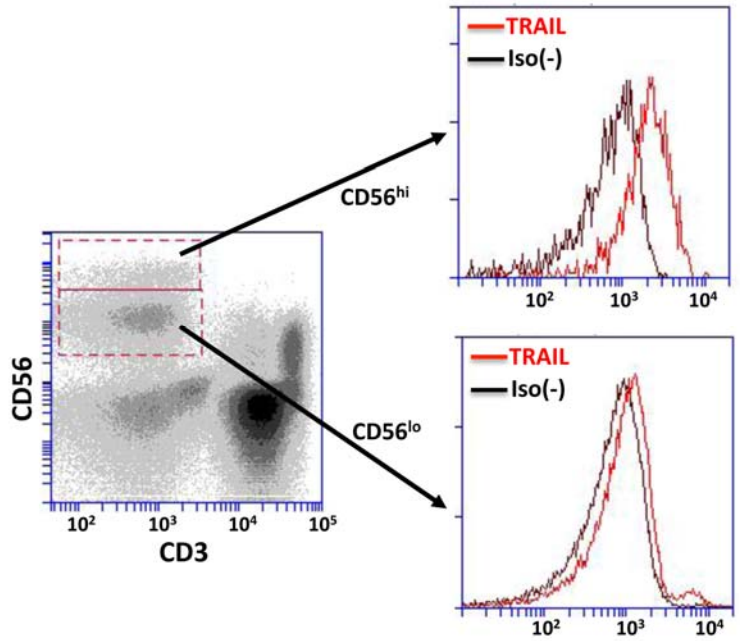

Figure S5. CD56^{hi} NK cells express high levels of TRAIL, Related to Figure 7. Freshly isolated human peripheral blood was analyzed by FACS to identify NK cells (CD3$^-$CD56$^+$), and were further assessed for TRAIL expression in CD56^{hi} and CD56^{lo} NK cell populations.

Table S1. Primers for construction of HCMV FIX mutants, Related to the Experimental Procedures

HCMV ∆UL139	UL139.for:<br>AGACGTCACTTT CACAATGACGTTC GTAGACACGTTG ATCATAAAACAC CCTACAAGGACG ACGACGACAAGT AA
	UL139.rev: GGTGTTTTATGA TCAACGTGTCTA CGAACGTCATTGTG AAAGTGACGTCTG TGACACAGGAACA CTTAACGGCTG A
HCMV ∆UL140	UL140.for:<br>ATGAACGTTC CTTCTTAAAC ATCCGAGGTA GCAATGAGAC AGGTCGCGTA
	UL140.rev:<br>TACGCGACCT GTCTCATTGC TACCTCGGATG TTTAAGAAGGA ACGTTCAT
HCMV ∆UL141	UL141.for CTGTTGAGC TGCCCGCGA CTCCTCGAAT ATTCTTCCTC TTCGTTCCCC TT
	UL141.rev TTCATCAGCC GATGGCCTTT GACGGCGTCC AGGATGAGCT CGTCGCTGCC GT
HCMV ∆UL139-UL141	UL139.for:<br>AGACGTCACT TTCACAATGACG TTCGTAGACACG TTGATCATAAA ACACC
	UL141.rev:<br>ATAACAACACG CCCATTCAATC CGCATATTTTAA TCACACTATTC ACAT

Supplemental Experimental Procedures

FACS. For intracellular UL44/p52 staining performed in figure S2, NHDF were permeabilized with a PBS 0.2% TX100-solution for 5 min at room temperature followed by incubation with anti-UL44 FITC 1μg/ml (clones CCH2+DDG9, Dako) at 4C° for 30min to 1hr. This was performed after staining for cell surface TRAIL DR levels.

Western blots. For western blots shown in figure S2, NHDF infected with HCMV FIX were incubated in the presence of cycloheximide (100 μg/ml; Sigma) at times 30 min prior to infection to 3h postinfection, followed by incubation in the presence of actinomycin D (2.5 μg/ml; Sigma) for another 3h. Selective expression of early genes was achieved by incubation of the cells in the presence of Foscarnet (250 μg/ml; Sigma) for the indicated times. Protein samples were taken from mock-infected cells and from infected cells at the indicated time points by lysis of the cells in protein sample buffer (3% SDS, 2% ß-mercaptoethanol, 200 mM Tris [pH 8.8], 0.5 M sucrose, 5 mM EDTA), and subsequently boiled for 5 min. Proteins were separated by sodium dodecyl sulfate-polyacrylamide gel electrophoresis (SDS-PAGE) (10% polyacrylamide) and transferred to nitrocellulose filters. Filters were probed with anti-UL141 antibody (Tomasec et al., 2005) (1:3000 dilution of M550.3 ascites), followed by secondary detection via goat anti-mouse HRP (Santa Cruz) respectively. Signals were detected by enhanced chemiluminescence with the ECL detection kit (Amersham).

Cell death assays. NHDF were seeded in 96 well dishes (1 x 10^4/well) and were infected the next day for 2 hours with HCMV (moi~2). Forty eight hours post infection, dilutions of FLAG tagged TRAIL were added in triplicate in media containing anti-FLAG antibody (2μg/ml) and cycloheximide (CHX, 5 μg/ml). Forty-eight hours later (96 total post infection) cell viability was determined using an MTT based assay, and percentage cell death was normalized to HCMV infected NHDF treated with CHX and anti-FLAG in the absence of ligand. For caspase 3/7 activation assays, HFF were seeded in 96 well dishes (1x10^4/well) and infected the next day with adenoviral vectors. Forty-eight hours post-infection, cells were treated with cycloheximide at 10μg/ml plus either TNF-α (30ng/ml, Sigma T6674) or TRAIL (150ng/ml, Sigma T9701). At the time of assay start, cell viability was determined on a replicate plate, using the WST-1 assay (Roche). CaspaseGlo 3/7 kit (Promega G8091) was used according to manufacturer's instruction to measure caspase activity. Relative luminescence units were normalized to cells treated with cycloheximide alone.

Plasmids and adenovirus. The mature ectodomain of UL141 (aa 37-279) was PCR amplified and cloned downstream of the human Ig signal sequence in PCR3-Fc. The UL141 ectodomain was also cloned in the pAcGP67A baculovirus transfer vector (BD biosciences) with the addition of an N-terminal his-tag (for use in Biacore). UL141 was cloned by amplification of the entire

coding sequence, including the stop codon, followed by cloning into the C1/Topo vector (Invitrogen). The hTRAIL-R2-GPI plasmid encodes the ectodomain of hTRAIL-R2 fused to a portion of hTRAIL-R3 that includes the GPI addition signal, and has been described previously (Bossen et al., 2006). For generating GFP and RFP tagged TRAIL-R2 constructs, pCR3-Flag-hTRAILR2 was used as a template (Bossen et al., 2006). TRAIL-R2 deleted for its entire death domain (DD) and C-terminus was fused to GFP or RFP at its C-terminal domain, generating RAd-TRAILR2ΔDD.GFP and RAd-TRAILR2ΔDeath Domain.RFP. Using pAL1065 containing MICA-YFP cDNA as a template (kind gift from D. Davis), MICA was tagged on its C-terminus with GFP, generating RAd-MICA.GFP. Using pAL880 containing CD155 cDNA as a template, CD155 was tagged on C-terminus with RFP, generating RAd-CD155.RFP. The ER marker-RFP was generated using the DsRed2-ER base plasmid (Clontech; 632409), and was recombineered into AdZ adenovirus vector. The ER-targeting sequence of calreticulin is fused to N-terminus of DsRed2 and the KDEL ER retention sequence is fused to the C-terminus. The Golgi marker-GFP was generated using the AcGFP1-Golgi base plasmid (Clontech; 632464), and was recombineered into AdZ adenovirus vector. AcGFP1 is fused at its N-terminus to 81 amino acids of human beta-1,4-galactosyltransferase, which contains the membrane anchoring signal peptide within this sequence that targets the fusion protein to the trans-medial Golgi apparatus.

SPR Analysis. SF9 cell purified UL141 was concentrated using an Amicon Centrifugal Filter Unit (Millipore, Ultracell-30K) while exchanging to Biacore running buffer (10 mM HEPES pH 7.4, 150 mM sodium chloride and 3 mM EDTA). After addition of 0.005% Tween 20, UL141 was immediately used for Surface Plasmon Resonance studies on a Biacore 3000 (GE Healthcare). Approximately 500 response units (RU) of hTRAIL-R1 and –R2:Fc were immobilized during two independent experiments on an anti-human Fc capture chip. Human LTβR:Fc was immobilized in both experiments on a second flow channel as a negative control (up to 500 RU). Serial dilutions of UL141 protein (0–50 μM and 0–1 μM) in Biacore running buffer were then injected for 4 min association, while dissociation was conducted over 10 and 30 min for TRAIL-R1 and -R2 kinetics, respectively. After each cycle, the chip was regenerated with a 30 sec injection of 2M $MgCl_2$ at 10μl/min and freshly coated with hTRAIL-R1 and –R2:Fc. Kinetic experiments were carried out at 25°C with a flow rate of 30 μl/min and repeated twice, each time with a different UL141, hTRAIL-R1 and –R2:Fc preparation. Kinetic parameters were calculated after subtracting binding to LTβR:Fc as a background, using a simple Langmuir 1:1 model in the BIA evaluation software version 4.1.

ELISA. For results shown in figure S4, UL141:Fc protein was coated in 100 mM Tris (1.5 μg/ml), pH 9.6 in 96 well Immuno plates (Nunc). Wells were blocked (1% BSA + 0.1% Tween in PBS) prior to addition of TRAIL-R1:Fc, TRAIL-R2:Fc or mLTβR:Fc diluted in blocking buffer. Samples were incubated for 1h at room temperature, washed 4X with wash buffer (1X PBS, 0.05% Tween) and

incubated with mouse anti-TR1 (eBioscience), mouse anti-TR2 (Alexis) or rat anti-LTβR clone 3C8 (De Trez et al., 2008), 5 μg/ml in block buffer for 1h. Wells were washed and incubated with either anti-mouse or anti-rat-HRP for 1 h at room temperature. After a final wash, bound HRP-antibodies were detected using TMB substrate (eBioscience) followed by 1M H_3PO_4 to stop. The absorbance at 450 nm was quantified for each well on a SpectraMax plate reader. The binding of UL141:Fc to the wells was confirmed using HRP-anti-human Fc specific antibody (Jackson Labs). Coating of TRAIL-R2:Fc or LTβR:Fc was done similarly, and binding of UL141:Fc was detected using a 1:2000 dilution of M550.3 ascites, followed by similar 2° antibodies and detection to that above.

NK killing assays. NK cells were isolated from donor PBMC (D44) by negative selection using antibody-coated bead separation (EasySep Enrichment Kits, StemCell Technologies) and cultured overnight in RPMI-1640 (Invitrogen) supplemented with 10% fetal calf serum, 1% pen/strep and 0.5% L-glutamine (Gibco) supplemented with 1000IU/ml IFN-α (Roferon, Roche). The following day, NK cells were washed and incubated with 10µg/ml of mIgG (Santa-Cruz), DNAM-1 (DX11, BD Pharmingen), sCD30 (RnD Sytems) and/or sTRAILR2 (Peptrotech) prior to mixing with $1x10^5$ CFSE loaded A549 cells which had been infected with RAdControl or RAdUL141 for 48hr at an MOI of 50 (E:T of 2:1). To control for background cell death, A549 cells were incubated in media only. Assays were carried out in 200µl round bottomed 96 well plates. After 4hr the supernatant was removed and the adherent targets were trypsinised (Gibco) and washed in PBS prior to the addition of 7AAD (Invitrogen Molecular Probes). Cells were analyzed by flow cytometry on a BD Accuri C6 flow cytometer (BD Biosciences). Target A549 cells were identified as CFSE positive and the percentage of $7AAD^{hi}$ target cells were determined by drawing a dot plot of FSC vs FL3. The percentage of $7AAD^{hi}$ cells in the test wells was calculated by subtracting the percentage of $7AAD^{hi}$ cells in the wells containing targets and media only. For analysis of TRAIL expression by IFNα activated NK cells, total donor PBMC were treated similarly, but after an overnight incubation cells were stained with CD3-FITC (MEM-57, Serotec), CD56-PE (N901, Beckman-Coulter) and TRAIL (RIK-2, eBioscience), followed by detection of RIK-2 binding with anti-mouse IgG AF647 (Invitrogen). Flow cytometry was performed on a BD Accuri C6 flow cytometer (BD Biosciences). NK cells were identified as being $CD3^-$ $CD56^+$.

Supplemental References

De Trez, C., Schneider, K., Potter, K., Droin, N., Fulton, J., Norris, P.S., Ha, S.W., Fu, Y.X., Murphy, T., Murphy, K.M., *et al.* (2008). The inhibitory HVEM-BTLA pathway counter regulates lymphotoxin receptor signaling to achieve homeostasis of dendritic cells. J Immunol *180*, 238-248.
